# Editorial: Progress in Ecological Stoichiometry

**DOI:** 10.3389/fmicb.2018.01957

**Published:** 2018-09-05

**Authors:** Dedmer B. Van de Waal, James J. Elser, Adam C. Martiny, Robert W. Sterner, James B. Cotner

**Affiliations:** ^1^Department of Aquatic Ecology, Netherlands Institute of Ecology (NIOO-KNAW), Wageningen, Netherlands; ^2^School of Life Sciences, Arizona State University, Tempe, AZ, United States; ^3^Flathead Lake Biological Station, University of Montana, Polson, MT, United States; ^4^Department of Earth System Science, University of California, Irvine, Irvine, CA, United States; ^5^Department of Ecology and Evolutionary Biology, University of California, Irvine, Irvine, CA, United States; ^6^Large Lakes Observatory, University of Minnesota Duluth, Duluth, MN, United States; ^7^Department of Ecology, Evolution and Behavior, University of Minnesota, Saint Paul, MN, United States

**Keywords:** biological stoichiometry, elemental composition, ecological theory, C:N:P, ecological scaling

## Introduction

Ecological Stoichiometry is the study of the balance of energy and multiple chemical elements in ecological interactions (Sterner and Elser, 2002). With a deep ancestry extending to J. Liebig and A. J. Lotka, during the twentieth century stoichiometric foundations were laid by A. C. Redfield, M. Droop, D. Tilman, W. Reiners, V. Smith, J. Urabe, and D. O. Hessen, among others. In their book, Sterner and Elser sought to assemble and articulate some of the core concepts of Ecological Stoichiometry. These include a formalized approach to variation in the strength of stoichiometric homeostasis among biota (weak in photoautotrophs, tight in consumers), extension of Urabe's Threshold Elemental Ratio (TER) approach, elucidation of the rules governing differential nutrient recycling by consumers, and organization of materials that lay the basis for the Growth Rate Hypothesis (GRH), linking the C:N:P stoichiometry of an organism to its growth rate due to the P-rich signature of the ribosomal growth machinery. Since the appearance of Sterner and Elser (2002), these core concepts of the stoichiometric framework have been extended widely. This is manifested in citation statistics. For example, as of 10 July 2018, Sterner and Elser (2002) has been cited a total of 3,728 times, including 566 citations since 2017 (Google Scholar). In Google Scholar, a search on *Ecological Stoichiometry* returns more than 40,000 records, with citations increasing at a ~8% rate annually during recent years.

Indeed, much has happened in the field of Ecological Stoichiometry in the past 15 or so years and an update is needed. The collection of papers in this Frontiers Research Topic is a cross-section of the diversity of applications of stoichiometric theory that have emerged. From this compilation we see how stoichiometric perspectives have been further applied in some areas (microbial processes and consumer-resource interactions), and extended in several novel directions to other areas, such as studies of host-pathogen interactions, of the role of pollen in trophic webs, and of fungi. While most stoichiometric work has focused on the core macroelements (C, N, and P), increasing attention is now being paid to other essential elements that play a role in organismal biology and ecosystem functioning. This broader view of the Periodic Table is seen in several papers in this collection. Importantly, in nearly all papers we see signs of the integrative thinking that stoichiometric theory catalyzes: the fact that chemical elements both make up the fundamental molecules of life and constitute one of the core foci of ecosystem ecology. This integration provides a means to create a seamless fabric across almost all of biology. We encourage readers to find their own common threads that connect these papers to their own research. Since pretty much everything that is a thing is, at its core, elemental, novel applications of the stoichiometric framework await.

## Microbes

A diverse set of papers are included in this Research Topic on microbial stoichiometry, spanning terrestrial to aquatic systems and including the human microbiome. Manzoni used a theoretical approach to follow C and N dynamics during litter degradation. Carbon use efficiency is flexible and increased under N depletion while decreased when N was replete. These findings suggest that decomposer community traits depend on litter stoichiometry and shift from N to C-limited conditions during decomposition. Zhang and Elser used a meta-analysis to examine the stoichiometry of fungi, a little-studied but incredibly diverse group. An important observation was that C content varied widely while N:P ratios were similar to the Redfield ratio. However, latitude and temperature affected N:P ratios while temperature also affected C:P ratios. In a study looking at microbial diversity in Cuatro Cienegas (Mexico), Lee et al. found that adding nutrients to this ultra-oligotrophic system disrupted community structure and promoted the growth of rare phototrophic species, while enriching at different N:P ratios had an impact above and beyond fertilization *per se*. In a paper examining temperature and nutrient effects on heterotrophic bacteria stoichiometry, Phillips et al. found that nutrients had a strong effect on stoichiometry and, perhaps more importantly, that temperature effects on stoichiometry were greatest when nutrient levels were lowest. At the ecosystem scale, They et al. observed that microbial biomass and the stoichiometry of dissolved pools diverged from each other in lakes with the longest residence times, suggesting that microbial influence on ecosystem stoichiometry is maximized at long residence times. In another ecosystem, humans, Vecchio-Pagan et al. used protein sequences to infer differences in microbial stoichiometry in the human microbiome. They observed that the skin and nares had higher N and O content while the gut microbiome was high in S. In a similar vein, Dittberner et al. found that open ocean microbial communities optimized the protein N use as an adaptation to low nutrient availability. Lastly, Linzner et al. examined the evolution of stoichiometry in *Escherichia coli*. They found that adaptation to increased temperature influenced the cellular stoichiometry but was mediated by the specific evolutionary pathway.

Although this collection of studies of microbes is diverse in approaches and habitats, these studies emphasize the important roles of temperature and nutrients in influencing microbial stoichiometry at different scales. The use of “omics” information to infer stoichiometry of communities will be an important tool going forward.

## Primary producers

The elemental composition of phytoplankton and other primary producers has been an area of intense research. Traditionally, the elemental stoichiometry of especially marine phytoplankton had been viewed as having limited variability (Redfield, 1958). Nonetheless, numerous physiological experiments have revealed strong stoichiometric responses to variations in light and nutrient availability (Geider and La Roche, 2002). However, several key questions are currently unanswered including the impact of producer diversity, the role of temperature as well as the interaction between temperature and nutrient limitation, and how changes in biochemistry is ultimately linked to overall cellular elemental composition (Moreno and Martiny, 2018). Several studies in this Research Topic aimed at addressing these three major unknowns.

Garcia et al. showed high variation in the elemental composition of marine eukaryotic phytoplankton, particularly within the group of Bacillariophyceae where variation was comparable to that among seven investigated classes. The authors tested different variables and showed that temperature accounted for a significant yet small portion of the variation. Yvon-Durocher et al. found that experimental as well as seasonal warming of freshwater ponds led to species sorting, nutrient drawdown, and increased C:P and N:P ratios of the community. Moreover, isolates of the green alga *Chlamydomonas* from the warm treatments exhibited higher C:P and N:P ratios, indicating that the direction and magnitude of stoichiometric shifts by local adaptation in response to warming were comparable to the overall shifts in seston stoichiometry. Velthuis et al. examined aquatic plants and showed, in support of theory, that nutrient addition consistently resulted in decreased carbon:nutrient ratios. However, elevated temperature did not change the elemental stoichiometry in a consistent manner. Villar-Argaiz et al. placed nutrient impacts within a broad context of multiple stressors including UV radiation, temperature, CO_2_, and others. Their main conclusion was that cellular compositional responses to these various stressors was most often non-additive, stressing the importance of context in exploring how ecological systems change over time. Using an elegant microplate factorial design, Hessen et al. also found that nutrient limitation had a relatively stronger impact than temperature on the elemental stoichiometry. However, the experiment also demonstrated how the macromolecular composition changed in response to both environmental factors. Changes in biochemical composition were similarly examined in Grosse et al. Here, it was shown that the cellular concentrations of amino acids, fatty acids and carbohydrates were sensitive to light and types of nutrient limitation. Fernandes et al. show how to use phytoplankton to effectively remove N and P from wastewater and found that P was assimilated faster than N. Thus, the stoichiometric requirement and nutrient uptake mechanisms are important to consider when using phytoplankton for wastewater treatment. The above-described studies demonstrate how diversity, temperature, and the biochemical regulation of macromolecules are important for understanding the elemental stoichiometry of producers.

## Herbivores

Studies on the plant-animal interface were central to the development of Ecological Stoichiometry and new insights continue to emerge. The plant-herbivore interaction is notable for its varying degree of stoichiometric mismatch. Sometimes primary producers offer a nutritious, elementally balanced forage for the next trophic level, and sometimes they are far from doing so. Studies specifically looking at controls and dynamics of element content and potential mismatches include Moody et al., who examined body composition and growth rate of two neotropical stream grazers (a mayfly and a tadpole) with an eye toward looking for indications consistent with the Growth Rate Hypothesis. Focusing on the temperature differences among streams at different elevations, the authors observed that among the mayflies, highest body P as well as highest growth occurred in the warmest, lowland stream, but overall the study emphasized that the GRH likely does not apply for temperature-based differences in growth. Most considerations of stoichiometric dimensions of food quality take a temporal perspective appropriate to assessing generation-long effects of plant element content on animal growth. However, studies are beginning to consider how to incorporate changing food within an animal's lifetime. Wagner et al. shifted *Daphnia magna* grazers from high to low P-content food and vice versa, and then looked at how body composition (P, RNA, ATP), and organism mass changed over several days following the shift. *Daphnia* did not react instantly, but they responded measurably to changing food conditions after 12–36 h. Changes in body P were first observed, then changes in RNA followed by changes in mass. These results help us understand how organisms integrate over variable food conditions.

Work continues to address the fundamental question of how much the biochemical forms of elements affect ecological dynamics. At its purest level, Ecological Stoichiometry is concerned only with elements, not with the forms they are found in. We all recognize that not all carbon or phosphorus atoms are equal, that in biological material some molecules are reactive while others are recalcitrant, etc., but the question for stoichiometrists is how much this biochemical variation, layered on top of elemental variation, matters. Zhou et al. report the interesting findings that P recently (< 90 min) incorporated by algae does not support growth as well as P that has been part of the algal cell for longer periods. Living beings are not bags of elements but we continue to ask how much we can understand with that simplified view. Two other contributions seek to understand how stoichiometric relationships revealed in one system carry over to other systems. Bracken reports on a geographic study with results that point to an importance of stoichiometric mismatch in rocky intertidal suspension feeders, and Sitters et al. point out that the vast literature on terrestrial herbivores has remarkably little to offer in terms of simultaneous analysis of N and P. There clearly is much yet to do to assemble relevant data sets to test stoichiometric theory in a wide diversity of habitats.

## Pathogens

Pathogens such as viruses and fungal parasites require nutrients from their hosts, and alterations in host stoichiometry may thus affect disease dynamics. Lacroix et al. studied the impact of plant stoichiometry on the infection dynamics of two viruses, by exposing the plant to a range of N:P supply ratios. Although nutrient supply and plant stoichiometry did not alter the titer of one of the viruses, infection by the other virus reduced the total titer, indicating within host nutrient competition. Moreover, higher nutrient supply rates affected host traits and caused an increase in infection and coinfection rates. Also the infection dynamics of a cyanobacterial fungal parasite were shown to depend on N:P supply ratios, as shown by Frenken et al. Their findings demonstrate how parasite N:P ratios follow that of their host, and increased with N:P supply. Moreover, production of parasite zoospores were shown to increase with host N:P ratios. It thus seems that these fungal parasites have relatively high N, but low P requirements. Together, these studies demonstrate how shifts in primary producer stoichiometry may alter infection dynamics of their pathogens, with possible consequences for higher trophic levels.

## Crossing scales

Ecological Stoichiometry spans a range of organizational scales. Cherif et al. describe an operational framework where they identify the processes connecting stoichiometry across a wide range of biological levels in order to characterize the consequences of stoichiometric imbalances at one level for all other levels. The review specifically highlights advances, potential for further development, and integration of theories from the genome to the biosphere level, including processes from gene expression to atmospheric and oceanic circulation of elements, thereby connecting all biological levels. At the organism level, the balance of carbon (energy) and nutrients is affected by traits. Meunier et al. cluster these traits in four main groups including acquisition, body stoichiometry, storage, and excretion. Their review provides a general description on the stoichiometry of traits, both in autotrophs and heterotrophs. Moreover, they highlight the role of trade-offs in determining the dominance of distinct traits in response to shifts in resource supply loads and ratios. Traits define the interactions between one organism and another, and between organisms and their environment. In turn, the environment determines the ecological niche of organisms. González et al. provide a multidimensional elemental view on this ecological niche, thus focusing on elemental niches rather than trait based niches. They propose an approach visualizing the stoichiometry of individuals in multivariate space in order to quantify niche dimensions within and between organisms. Their analysis integrates stoichiometric niches occupied by terrestrial and freshwater food webs, trophic groups, individual species, and individuals with species. With complementarity tests, this method allows the assessment of unoccupied stoichiometric niche space.

Ecological Stoichiometry can also be applied for connecting ecology with biogeochemistry and ecosystem metabolism, which is highlighted by Welti et al. They describe how trophic interactions (and nutrient requirements) link biogeochemistry to food webs, how carbon: nutrient ratios (and nutrient limitation) link food webs to ecosystem metabolism, and how elemental fluxes and transformation rates link ecosystem metabolism to biogeochemistry. Global fluxes of N and P have been perturbed over the last century, and Guignard et al. explore the impacts of these changes on the stoichiometry and genomic traits of organisms. Responses of organisms to changes in N and P loads and ratios may depend on their genome size, as larger genomes have higher N and P demands, yet genome size is an often overlooked trait. Importantly, the authors highlight that we are close to the planetary boundary of a safe operating space for P flowing into the ocean (Rockström et al., 2009), which calls for a more sustainable use of fertilizers. To this end, applying Ecological Stoichiometry to agricultural practices may enable the maintenance of agricultural productivity, while conserving biodiversity and thereby supporting the wide range of services that ecosystems provide across the world.

Organisms can transport elements between different environments. Luhring et al. developed a framework to describe the stoichiometry of elemental fluxes among ecosystems. Specifically, they describe how life histories of amphibians and salmon determine the relative fluxes of elements between freshwater-terrestrial and freshwater-marine ecosystems, respectively. Their work shows how fluxes may differ between elements and depend on life-history, leading to simultaneous imports and exports of different elements depending on ontogeny and the movement of organisms between systems. Cross-system transfer of elements not only occurs via animals, but also by plants. Indeed, Filipiak describes how temporal nutrient limitation by detritivores may be alleviated by pine pollen, in both terrestrial but also aquatic ecosystems. Compared to litter, pollen is relatively balanced in nitrogen and phosphorus and contains a range of additional valuable elements. Consequently, pollen rains may substantially add nutrients to ecosystems, particularly aquatic ones. Thus, pollen may play an important role in nutrient cycling both within and across ecosystems. The stoichiometry of decomposition may differ from that of primary production. Indeed, Evans-White and Halvorson describe how detritus based “brown” aquatic food webs differ from autotroph-based “green” food webs with respect to C quality and nutrient contents. In a meta-analysis, they show how N and P availabilities largely limit both detritivores and herbivores following general stoichiometric principles, but also show distinct differences in the mechanisms of limitation due to distinct consumer regulatory processes in both types of food webs. Together, these studies demonstrate how Ecological Stoichiometry can be used to connect a wide range of scales, from subcellular processes to ecosystem dynamics and services.

## Beyond C:N:P

Since the days of Redfield (1958), stoichiometric approaches have had a predominant focus on three elements: C, N, and P. This makes sense, as C is the architectural linchpin of biomass and a very useful proxy for tracking energy, while N and P are key constituents of vital biomolecules (protein and nucleic acids) and often limiting to biota of all kinds. However, more than 2–3 dozen elements are essential for living things and a number of these can also be limiting to different biota at various times and under various conditions. So, it would make sense to extend stoichiometric thinking to other essential elements in the Periodic Table. Such efforts are reflected here. Karl and Grabowski bring attention to a much-neglected element, hydrogen (H), perhaps the “plain brown wrapper” of the elements: ubiquitous in biota, an essential regulator of organic matter redox state, and, while frequently measured in CHN analyzers, generally ignored. Karl and Grabowski call for more accurate determinations of the H content of organic matter and argue that such data will yield better estimates of the energy state of organic matter and thus its impacts in ecosystems. Jeyasingh et al. move to broad swaths of the Periodic Table, arguing for an “ionomic” perspective, highlighting key tradeoffs that exist because of the coupling of elements in biological processes. Evidence is presented for characteristic and coupled shifts in sets of chemical elements. For example, Mg, Na, and K were seen to be associated with each other in experimental studies of numerous strains of freshwater bacterial heterotrophs grown under different conditions of P supply. Much work remains to illuminate these complex interactions and Jeyasingh et al. sketch out some promising paths forward.

Of course, elements are used to make molecules and the relative balance of major molecules (e.g., proteins, carbohydrates, lipids) and essential biochemical components (e.g., essential fatty acids) can also play an important role in determining the nutritional quality of food. Often similar analytical and conceptual frameworks can be used for analyzing such biochemical dimensions as are used in elemental stoichiometry studies. Anderson et al. take such an approach to detritus-based food webs in the ocean, analyzing “trophic upgrading” of C:PUFA (polyunsaturated fatty acids) ratios by bacteria and their protist consumers, pointing to a complex interplay between the abundance of food and its quality in supporting mesopelagic copepods.

## Outlook

The field of Ecological Stoichiometry has matured considerably in the 15 years or so since the publication of Sterner and Elser (2002), which is evident from the studies presented in this Research Topic. This collection of papers covers the application of Ecological Stoichiometry in a wide range of topics, from microbes, to primary producers, herbivores, pathogens and entire ecosystems, that are tied together by Ecological Stoichiometry alone, or coupled to existing ecological frameworks, including C, N, and P, as well as the remaining Periodic Table. Ecological Stoichiometry was even shown to have societal relevance by application in agriculture, wastewater treatment and assessing ecosystem services. Clearly, there is still a lot more work that needs to be done! The presented papers not only provide a tremendous infilling of facts and details, but also extend in new exciting areas.

Novel areas where we see that Ecological Stoichiometry can and most likely will contribute substantially in the future include, for instance, human health via food nutrition (Myers et al., 2014), and the understanding of disease virulence or suppression in the human microbiome (Bäumler and Sperandio, 2016). In agriculture, better understanding of the coupling of nutrient cycles is and will be increasingly important for sustainable food production, with simultaneous benefits toward ecosystem services. Relatedly, it is worth mentioning that humans are conducting a huge stoichiometric experiment through our manipulation of the carbon cycle, with important implications to all living things (Loladze, 2002). Moreover, with an eye toward balancing production and recovery of elements in waste streams, we may more effectively close multiple elemental cycles. There are clearly many more areas where Ecological Stoichiometry will provide useful contributions, both in our basic understanding of ecosystems, as well as applications to societal issues. As the papers assembled in this collection demonstrate, Ecological Stoichiometry continues to provide a useful lens with which to study the world with nature's infinite complexity. Sterner and Elser's tome presented a huge scaffolding for understanding macromolecules, organisms, communities and ecosystems in light of Ecological Stoichiometry. The details of what lies within that framework are incredibly fascinating and will continue to be important, interesting and relevant for a long time.

## Author contributions

All authors listed have made a substantial, direct and intellectual contribution to the work, and approved it for publication.

### Conflict of interest statement

The authors declare that the research was conducted in the absence of any commercial or financial relationships that could be construed as a potential conflict of interest.
